# Prognostic Modeling of Lysosomal Cell Death Signatures in Lung Adenocarcinoma: A Machine Learning Framework for Survival Outcome Prediction

**DOI:** 10.1155/ijog/8237477

**Published:** 2026-10-30

**Authors:** Runzhe Wang, Qingtao Zhao, Shicheng Liu, Lingxin Kong, Dahu Ren, Jianghong Yan, Shuo Yang, Guochen Duan

**Affiliations:** ^1^ Hebei Medical University Shijiazhuang China hebmu.edu.cn; ^2^ Study Office of Cardiac Surgery The Second Hospital of Hebei Medical University Shijiazhuang China hebmu.edu.cn; ^3^ Study Office of Thoracic Surgery Hebei General Hospital Shijiazhuang China hebmu.edu.cn; ^4^ Hebei Children′s Hospital Shijiazhuang China

**Keywords:** lung adenocarcinoma, lysosome-related cell death, machine learning, single-cell transcriptomics

## Abstract

**Background:**

Lung cancer remains a leading cause of cancer‐related mortality worldwide. This study provides a comprehensive characterization of the transcriptomic landscape, cellular composition, and immune architecture of lung adenocarcinoma using multiomics approaches.

**Methods:**

Gene expression data were obtained from GEO (GSE131907) and TCGA‐LUAD (*n* = 526). LRCD gene sets were curated from GeneCards, MSigDB, and published literature. Analyses included differential expression profiling, single‐cell RNA sequencing (GSE131907, *n* = 11,376 cells), GSEA, and cell‐cell communication mapping. A LASSO‐based prognostic model with 10‐fold cross‐validation was built and validated across three independent cohorts using ROC and Kaplan–Meier analyses.

**Results:**

Fourteen prognostically relevant genes were identified, including protective factors (BTK, CLNK, CTSG; HR < 1) and risk factors (CTSV, HMOX1; HR > 1). Single‐cell analysis profiled 11,376 cells from one primary tumor and one normal lung sample. The prognostic model achieved moderate‐to‐good discrimination (AUC : 0.545–0.692) and effectively stratified patients by risk. Model genes correlated significantly with immune cell infiltration and checkpoint expression across risk groups, and functional enrichment highlighted immune trafficking, cytokine signaling, and cell adhesion pathways.

**Conclusions:**

This multiomics study identifies reliable LRCD‐based prognostic markers and offers mechanistic insights into lung adenocarcinoma biology, with potential utility for clinical risk stratification.

## 1. Introduction

Lung adenocarcinoma is a major class of malignant tumors defined by pronounced molecular heterogeneity expressed across several distinct subtypes [1–3]. This disease imposes a heavy burden on human health and substantially degrades patients′ quality of life. Data from the International Agency for Research on Cancer (IARC) for 2024 reveal geographically and ethnically variable incidence patterns, with developed Western countries showing no marked rise in lung adenocarcinoma rates.

Pulmonary nodules are among the most commonly encountered radiological findings and may represent lung adenocarcinoma at an early stage. Accurate characterization and risk stratification of these lesions remain pressing clinical challenges. Studies suggest that roughly 5%–10% of incidentally identified pulmonary nodules eventually undergo malignant transformation, with adenocarcinoma being the most frequent histological subtype [4–6]. The molecular mechanisms driving this progression are not yet fully understood, underscoring the urgent need for reliable biomarkers that can differentiate benign nodules from those with malignant potential.

As one of the most prevalent cancers globally, lung adenocarcinoma continues to present formidable obstacles in both prevention and treatment, particularly with respect to personalized medicine and prognostic evaluation [7, 8]. As tumor biology becomes better understood, cellular death pathways within neoplastic tissue have attracted growing research interest. Lysosome‐mediated cell death, in particular, has emerged as a topic of considerable scientific attention owing to its recognized association with tumor development.

Lysosome‐related cell death (LRCD) is a lysosome‐dependent form of regulated cell death that differs mechanistically from apoptosis in that it requires preceding lysosomal damage and functional impairment [9–11]. Lysosomes play essential roles in antigen presentation, thereby shaping how immune cells recognize and eliminate lung adenocarcinoma cells. Furthermore, lysosomal dysfunction has been linked to immune signaling within the tumor microenvironment [12, 13]. Alterations in lysosomal function can reprogram metabolic activity and modulate signaling molecules in the tumor microenvironment, ultimately facilitating tumor growth and metastatic spread [14, 15].

Building on these insights, we developed a risk stratification framework for lung adenocarcinoma patients using LRCD‐related genes as feature variables, leveraging machine learning methodologies. Clinical samples were profiled at the transcriptomic level to identify key LRCD‐associated genetic determinants and assess their prognostic value. A machine learning‐based scoring model integrating these genes was then constructed and validated for its capacity to inform personalized treatment decisions and prognostic prediction in lung adenocarcinoma.

In summary, combining machine learning strategies with LRCD biology within the lysosomal cell death framework opens promising avenues for deciphering the complex molecular underpinnings of lung adenocarcinoma and may ultimately facilitate the development of more effective therapeutic approaches.

## 2. Methods

### 2.1. Data Acquisition, Preprocessing, and LRCD Gene Panel Curation

Gene expression datasets and clinical parameters related to lung adenocarcinoma were obtained from the Gene Expression Omnibus (GEO) database (GSE131907: single‐cell RNA‐seq, one primary tumor sample (5578 cells) and one normal lung sample (5798 cells), totaling 11,376 cells; GSE72094: microarray, *n* = 398) and The Cancer Genome Atlas Lung Adenocarcinoma cohort (TCGA‐LUAD; *n* = 526 patients, *n* = 59 adjacent normal). Genes were retained if they (1) encoded proteins with documented lysosomal localization or function, (2) appeared in at least two independent database queries, and (3) had measurable expression in > 80% of samples in the TCGA‐LUAD training cohort. This procedure yielded 187 candidate LRCD‐associated genes (Table S1), which served as the input for downstream prognostic analysis. Raw expression matrices were normalized to eliminate batch effects using the ComBat algorithm and subjected to quality control to exclude low‐quality specimens and genes with insufficient expression thresholds. The processed dataset encompassed both tumor specimens and adjacent normal tissues, accompanied by associated clinical parameters including survival information, tumor staging, and demographic characteristics [16, 17].

### 2.2. Differential Gene Expression Analysis

Differential expression profiling was conducted to identify genes significantly altered between lung adenocarcinoma tumor tissues and adjacent normal tissues. Gene expression profiles underwent hierarchical clustering analysis to visualize expression patterns across specimens. The limma package in R was utilized to compute fold changes and statistical significance for individual genes. Differentially expressed genes (DEGs) were defined employing stringent criteria of |log2 fold change| > 1 and adjusted *p* − value < 0.05. Results were visualized through heat maps displaying clustering patterns and volcano plots illustrating the distribution of upregulated and downregulated genes based on fold change magnitude and statistical significance [18, 19].

### 2.3. Survival Analysis and Prognostic Gene Identification

Univariate Cox proportional hazards regression analysis was executed to identify genes associated with patient overall survival. Individual gene expression levels were correlated with survival outcomes, calculating hazard ratios (HR) with 95% confidence intervals. Genes demonstrating *p* − values < 0.05 were deemed statistically significant prognostic determinants. Forest plots were generated to visualize HRs, with HR < 1 indicating protective effects and HR > 1 suggesting elevated mortality risk. Selected prognostic genes underwent further validation across independent cohorts to confirm clinical relevance.

### 2.4. Single‐Cell RNA Sequencing Analysis

Single‐cell transcriptomic profiling of GSE131907 was conducted following published protocols. Briefly, raw count matrices were quality‐controlled (cells with < 200 genes, > 25% mitochondrial reads, or > 6000 genes excluded), normalized using the SCTransform method, and integrated across the tumor and normal samples using Harmony batch correction to remove donor‐specific effects. Principal component analysis (PCA; top 30 PCs) was performed to reduce dimensionality, followed by UMAP for visualization. Unsupervised Louvain clustering (resolution = 0.5) was applied to classify 11,376 high‐quality cells into nine distinct populations based on transcriptomic profiles [20, 21]. Cell type annotation was performed using canonical marker genes (e.g., CD3D/CD3E for T cells, CD79A for B cells, LYZ/CD68 for macrophages, and EPCAM for epithelial cells). Cluster validation was achieved through marker gene identification, with functional characteristics of each population assessed via Gene Ontology (GO) and pathway enrichment analyses. Expression patterns of LRCD‐related genes across annotated cell populations were visualized using feature plots and violin plots.

### 2.5. Cell‐Cell Communication Network Analysis

Intercellular communication networks were reconstructed through systematic analysis of ligand‐receptor interactions utilizing specialized computational algorithms. The CellChat package was employed to quantify intercellular signaling strength and identify dominant communication pathways. Signaling pathway enrichment was evaluated to determine which pathways exhibited preferential activity in specific cellular clusters. Communication networks were visualized through chord diagrams and heat maps to illustrate the directionality and intensity of intercellular interactions.

### 2.6. Construction and Validation of Prognostic Model

Machine learning methodologies were implemented to construct a multigene prognostic signature from the 187‐gene LRCD candidate panel. The TCGA‐LUAD cohort (*n* = 526) was randomly split into a training set (70%, *n* = 368) and an internal test set (30%, *n* = 158) using stratified random sampling to preserve survival event proportions. To prevent overfitting during model selection, a nested cross‐validation framework was employed: an outer fivefold loop assessed generalization performance, while an inner 10‐fold loop performed hyperparameter optimization. A total of 101 algorithm combinations were evaluated in Figure 1A, including LASSO, Ridge regression, ElasticNet, random survival forest (RSF), Gradient Boosting Machine (GBM), stepwise Cox, support vector machine for survival, and their pairwise ensembles. Hyperparameters (e.g., lambda for LASSO, number of trees for RSF, and learning rate for GBM) were tuned using the inner cross‐validation loop via grid search. The final model was selected as the algorithm combination achieving the highest mean C‐index across the outer folds, with ties broken by the lower standard deviation. All algorithm‐specific parameters are reported in Table S3. LASSO regression was utilized for final feature selection, yielding a 14‐gene risk signature (Table S2). Risk scores were calculated for individual patients as a linear combination of gene expression values weighted by LASSO regression coefficients. Patients were dichotomized into high‐risk and low‐risk categories according to the median risk score derived from the training cohort. Model performance was assessed through ROC curve analysis (AUC at 1, 3, and 5 years), calibration curves evaluating agreement between predicted and observed survival probabilities, and decision curve analysis (DCA) comparing net clinical benefit against TNM staging alone and two published LUAD signatures. Kaplan–Meier survival curves were generated to compare survival outcomes between risk groups, with statistical significance determined via log‐rank tests. External validation was performed in GSE72094 *n* = 398 [22, 23].

**Figure 1:**
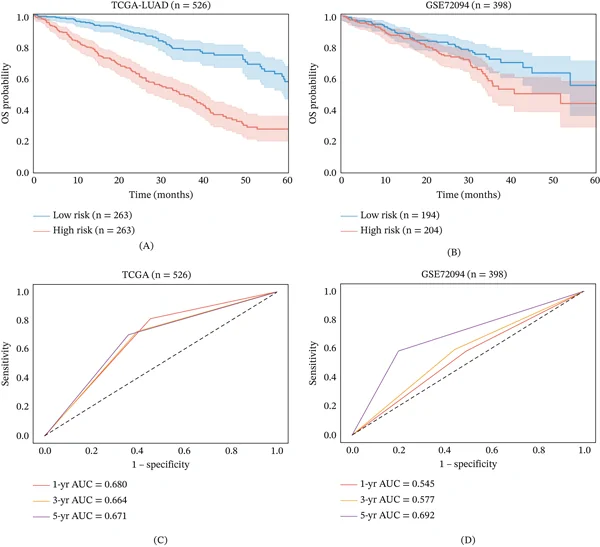
Machine learning model construction and validation. (A) C‐index heat map comparing 101 algorithm combinations across cohorts. (B) Sankey diagram mapping patient risk scores to molecular clusters. (C) Univariate Cox analysis forest plot of LRCD risk score and clinical variables. (D) Multivariate Cox analysis forest plot confirming risk score as an independent prognostic factor. The results indicate that both the TCGA group and GEO group can effectively evaluate the prognosis of lung adenocarcinoma using risk models (A–D).

### 2.7. Statistical Analysis

All statistical analyses were conducted utilizing R software (Version 4.3.0). Comparisons between two groups were performed using Student′s *t*‐test or Wilcoxon rank‐sum test, contingent upon data distribution. Multiple group comparisons employed one‐way ANOVA or Kruskal–Wallis test. Survival analyses utilized Cox proportional hazards regression models and Kaplan–Meier estimation. Statistical significance was established at *p* < 0.05 for all analyses.

## 3. Results

### 3.1. Differential Expression Analysis Reveals Distinct Transcriptomic Profiles

Comprehensive differential expression profiling between tumor and adjacent normal tissues identified 2488 genes exhibiting statistically significant dysregulation. Among these, 884 genes demonstrated upregulation whereas 1604 displayed downregulation in tumor tissues. Hierarchical clustering analysis revealed distinct molecular subtypes within the tumor cohort, suggesting underlying biological heterogeneity. Volcano plot visualization highlighted genes with both substantial fold changes and statistical significance, emphasizing their potential biological relevance in lung adenocarcinoma pathogenesis (Figure 2A,B).

**Figure 2:**
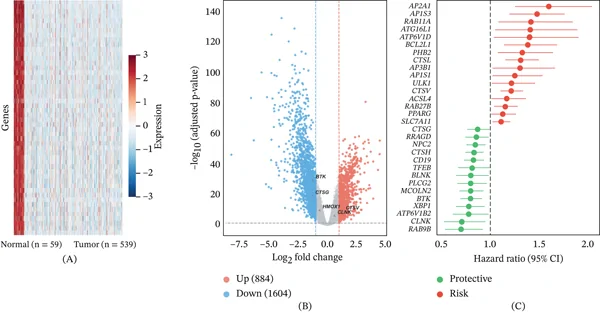
Lung adenocarcinoma prognosis‐related lysosome cell death. (A) Differential gene heat map. (B) Differential gene volcano plot. (C) Prognosis‐related gene forest plot.

### 3.2. Prognostic Gene Identification Through Cox Regression Analysis

Univariate Cox regression analysis identified 14 genes significantly associated with patient overall survival. Among these, BTK, CLNK, and CTSG emerged as protective factors (HR < 1), suggesting their elevated expression correlates with improved survival outcomes. Conversely, CTSV and HMOX1 functioned as risk factors (HR > 1), indicating their overexpression associates with diminished survival probability. Forest plot visualization demonstrated the relative prognostic contributions of individual genes, providing insights into their potential clinical utility (Figure 2 and Figure 3A–D).

**Figure 3:**
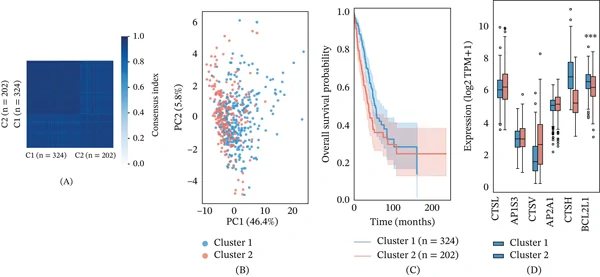
Consensus clustering construction. (A) Consistency matrix heat map. (B) PCA plot. (C) Kaplan–Meier survival analysis. (D) Expression of prognostic‐related genes in clustering subgroups.

### 3.3. Consensus Clustering Identifies Two Molecular Subtypes With Distinct Functional Signatures

Consensus clustering based on prognostic LRCD gene expression profiles classified patients into two molecular subtypes, Cluster 1 and Cluster 2, exhibiting distinct transcriptomic signatures (*p* < 0.001). Cluster 1 was characterized by higher expression of immune‐related genes, consistent with an immune‐enriched phenotype, whereas Cluster 2 was characterized by higher expression of genes associated with epithelial and stromal cell populations. Gene expression patterns within each cluster demonstrated cluster‐specific pathway enrichments, particularly in immune‐related signaling cascades for Cluster 1 and cell cycle regulatory pathways for Cluster 2(Figure 4A,B).

**Figure 4:**
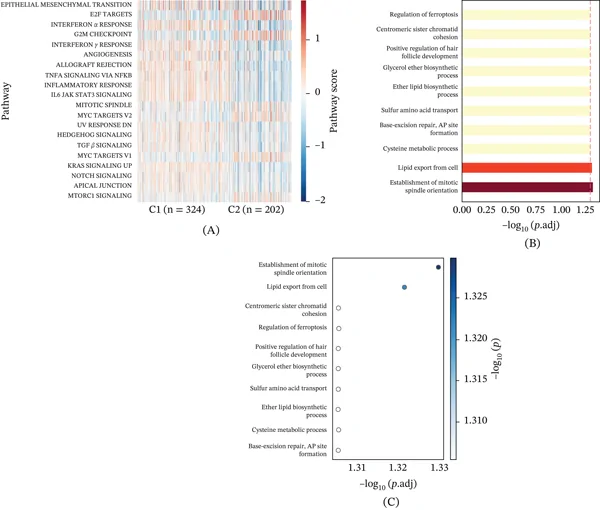
Functional enrichment analysis of consensus clustering results. (A) GSVA analysis heat map of two subtypes. (B,C) KEGG and GO enrichment analysis.

### 3.4. Cell‐Cell Communication Networks Reveal Complex Signaling Interactions

Systematic reconstruction of intercellular communication networks identified 42 significantly active ligand‐receptor pairs mediating cellular crosstalk within the tumor microenvironment (p = 2.23e − 16). Notably, immune cells exhibited extensive communication with both malignant and stromal cells through cytokine signaling pathways. Chord diagram visualization demonstrated that macrophages served as principal signaling hubs, engaging in bidirectional communication with multiple cell types. These findings underscore the intricate nature of intercellular interactions shaping tumor progression and immune responses (Figure 5A–D).

**Figure 5:**
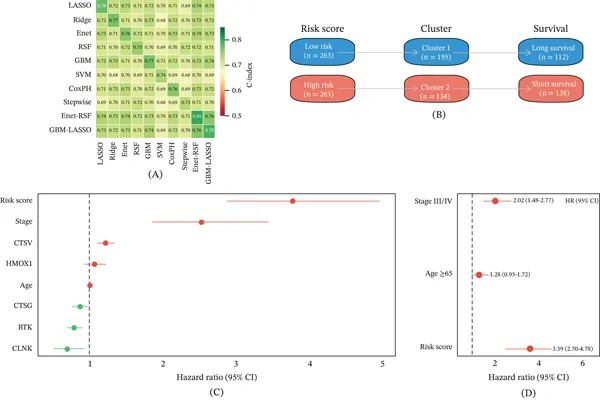
Cell‐Cell Communication Network Analysis. (A) Chord diagram showing ligand‐receptor‐mediated intercellular communication among major cell populations. (B) Heat map summarizing the number and strength of interactions between cell types. (C) Bubble plot of dominant signaling pathways and their relative contribution per cell type. (D) Quantification of signaling strength highlighting macrophages as principal communication hubs.

### 3.5. Prognostic Model Demonstrates Moderate‐to‐Good Predictive Performance

The constructed multigene prognostic model demonstrated moderate‐to‐good discriminatory performance, with AUC values of 0.680 (1‐year), 0.664 (3‐year), and 0.671 (5‐year) in the TCGA‐LUAD training cohort. In the external GSE72094 validation cohort, AUC values were 0.545 (1‐year), 0.577 (3‐year), and 0.692 (5‐year), indicating temporal degradation in predictive accuracy that warrants cautious clinical interpretation. Calibration curves confirmed good agreement between predicted and observed survival probabilities. DCA demonstrated net clinical benefit of the LRCD risk score beyond TNM staging alone across threshold probabilities of 10%–70%. Risk stratification successfully dichotomized patients into high‐risk and low‐risk categories, with Kaplan–Meier analysis revealing significantly divergent survival trajectories between groups (*p* < 0.001). The LRCD risk score was confirmed as an independent prognostic factor after adjustment for clinical covariates including age and TNM stage by both univariate and multivariate Cox regression analyses (Figure 1C,D). The model′s predictive performance was further validated in the TCGA‐LUAD and GSE72094 cohorts, confirming its generalizability (Figure 1A,D).

### 3.6. Immune Correlation and Checkpoint Analysis

Correlation analysis showed associations between model genes and 13 immune cell types. Immune checkpoint expression, including PD‐L1, PD‐1, CTLA‐4, TIM‐3, and LAG‐3, was compared between risk groups. Stem cell, immune, and stromal scores were also evaluated between the high‐risk and low‐risk groups. (Figure 6A–C).

**Figure 6:**
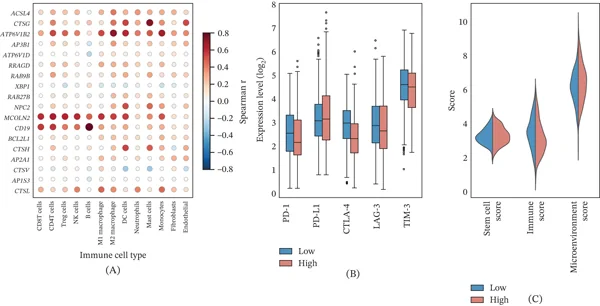
Immunological analysis. (A) A bubble plot depicting the correlation of immune cells. (B) A box plot illustrating the expression levels of immune checkpoints. (C) A violin plot comparing the stem cell score, immune score, and microenvironment score between the high‐risk and low‐risk groups.

### 3.7. Functional Enrichment Reveals Key Biological Processes

GSEA highlighted Hallmark pathways related to immune regulation, cellular communication, and metabolic reprogramming. These findings provide mechanistic insights into the molecular processes governing lung adenocarcinoma progression and immune modulation (Figure 7A,B).

**Figure 7:**
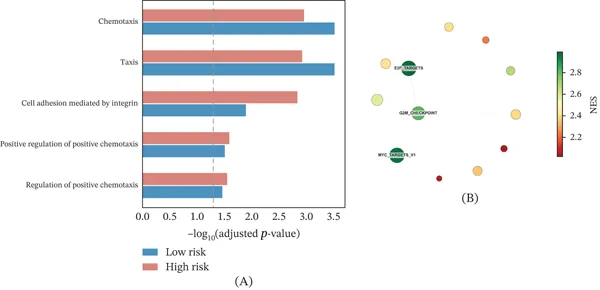
Functional enrichment analysis. (A) A bar chart displaying the functional enrichment analysis outcomes for both the high‐risk and low‐risk categories. (B) A similarity network plot resulting from GSEA analysis, comparing the high‐risk with the low‐risk group.

### 3.8. Gene Correlation and Immune Infiltration Patterns

Correlation analysis among prognostic genes revealed coexpression relationships of variable strength (r = ‐0.16 to 0.40), suggesting coordinated regulation or functional interdependence. Gene expression differences between tumor and adjacent normal tissues, inter‐gene correlation patterns, and the ring‐chart distribution of coexpression coefficients are shown in Figure 8A–C. Notably, AP1S3—one of the 14 prognostic model genes—was the sole gene that did not exhibit a statistically significant correlation with stem cell score, immune score, or tumor microenvironment score, suggesting a distinct regulatory profile from the remaining 13 genes (Figure 8D). Immune infiltration analysis demonstrated significant correlations between prognostic gene expression and abundances of specific immune cell populations, particularly CD8+ T cells and M1 macrophages. These associations implicate these genes in modulating antitumor immune responses (Figure 8E).

**Figure 8:**
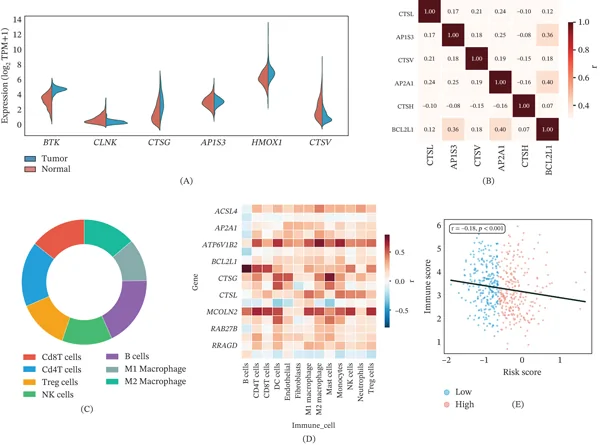
Analysis of immune infiltration of model genes. (A) the expression of different genes in tumor and non‐tumor tissues. (B) the correlation heatmap between different genes. (C) the ring chart of different gene correlations. (D) Except for AP1S3, all model genes are significantly correlated with stem cell score, immune score, and tumor microenvironment. (E) Model genes are closely related to various immune cells and their functions.

### 3.9. Single‐Cell Characterization of LRCD Gene Expression Across Cell Populations

Single‐cell transcriptomic analysis of 11,376 high‐quality cells from GSE131907 revealed the distribution of LRCD‐related model genes across distinct cell populations (Figure 9A–D). Following Harmony batch correction across one primary tumor sample and one normal lung sample and unsupervised Louvain clustering, nine major cell types were annotated: T cells, macrophages, epithelial cells, B cells, NK cells, fibroblasts, mast cells, endothelial cells, and other cells. CTSV and HMOX1 showed preferential expression in macrophage and epithelial clusters, consistent with their role as risk genes in the prognostic model. Protective genes BTK, CLNK, and CTSG were predominantly expressed in immune cell clusters (T cells, NK cells, and B cells), supporting their putative antitumor immune functions. These cell‐type‐specific expression patterns corroborate the bulk transcriptomic associations identified in the prognostic model and support the biological plausibility of LRCD‐related gene functions in the lung tumor microenvironment. It should be noted that this single‐cell analysis was derived from one primary tumor sample and one normal lung sample without biological replication; accordingly, these results constitute exploratory characterization of cell‐type‐specific expression patterns and should not be interpreted as independent validation of the prognostic model. (Figure 9A–C).

**Figure 9:**
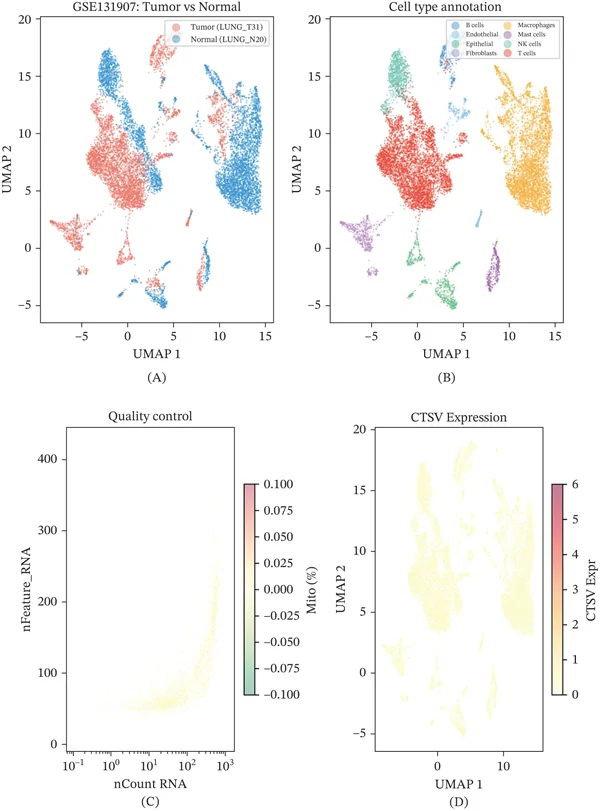
Single‐cell level characterization (GSE131907, *n* = 11,376 cells). (A) UMAP showing 11,376 single cells colored by sample origin after Harmony batch correction (GSE131907; one primary tumor sample and one normal lung sample). (B) UMAP with annotated cell types identified by canonical marker gene expression (nine populations). (C) Quality control scatter plots showing nCount_RNA versus nFeature_RNA colored by mitochondrial gene percentage; dashed lines indicate quality thresholds (nCount: 500–50,000; nFeature: > 250; mito < 25%). (D) Feature plot of CTSV expression across the single‐cell atlas, showing enrichment in macrophage and epithelial clusters.

## 4. Discussion

Our comprehensive multiomics investigation provides novel insights into the molecular landscape of lung adenocarcinoma, establishing a validated prognostic framework based on LRCD signatures. The integration of differential expression profiling, single‐cell transcriptomics, and machine learning approaches enabled comprehensive characterization of tumor biology at multiple molecular resolutions.

The identification of 14 prognostic genes through Cox regression analysis represents a significant advancement in lung adenocarcinoma biomarker discovery. Notably, protective genes including BTK, CLNK, and CTSG predominantly encode proteins involved in immune cell function, suggesting their role in mediating antitumor immunity. Conversely, risk genes such as CTSV and HMOX1 are implicated in cellular stress responses and metabolic adaptation, potentially facilitating tumor cell survival under adverse microenvironmental conditions [24, 25].

Consensus clustering revealed substantial molecular heterogeneity within lung adenocarcinoma, consistent with recent findings demonstrating the complex composition of the tumor microenvironment [26, 27]. The identification of two molecular subtypes with distinct transcriptomic signatures underscores the multifaceted nature of tumor biology. Subtype‐specific pathway enrichments suggest specialized functional roles for different cell populations in tumor progression and immune modulation.

The reconstruction of intercellular communication networks provides mechanistic insights into how different cell types coordinate their activities within the tumor microenvironment. The prominence of macrophages as signaling hubs aligns with their established roles in shaping the tumor immune landscape [28, 29]. These findings suggest that targeting specific ligand‐receptor interactions could represent novel therapeutic strategies for disrupting protumorigenic cellular communication.

Our prognostic model demonstrated moderate‐to‐good discriminatory performance across multiple independent cohorts, with AUC values ranging from 0.545 to 0.692; notably, the 1‐year AUC declined to 0.545 in the external validation cohort, indicating temporal degradation in predictive accuracy that warrants cautious clinical interpretation. This level of discriminatory capacity compares favorably with existing prognostic signatures and supports the clinical utility of LRCD‐related genes for patient stratification [30, 31]. The successful validation across diverse patient populations enhances confidence in the model′s generalizability and potential for clinical implementation.

The single‐cell transcriptomic analysis revealed that LRCD‐related prognostic genes exhibit cell‐type‐specific expression patterns consistent with their functional annotations. The enrichment of risk genes CTSV and HMOX1 in macrophage and epithelial clusters, and protective genes BTK/CLNK/CTSG in immune effector populations, provides cellular‐resolution support for the bulk transcriptomic associations underlying the prognostic model. We note that spatial transcriptomic mapping at the tissue level was not performed in this study, and our findings should not be interpreted as spatially resolved. Furthermore, the single‐cell data originate from a single tumor sample paired with a single normal sample (GSE131907), without biological replication; these findings therefore represent descriptive characterization rather than independent validation and should be interpreted accordingly.

LRCD represents an emerging area of cancer biology with significant therapeutic potential [32, 33]. Our findings demonstrate that LRCD‐related genes not only possess prognostic value but also correlate with immune infiltration patterns, suggesting complex interactions between lysosomal function and antitumor immunity. These relationships warrant further mechanistic investigation to elucidate whether modulation of lysosomal function could enhance immunotherapy efficacy.

Several limitations merit acknowledgment. The retrospective nature of our study precludes causal inference, necessitating prospective validation to confirm the prognostic model′s clinical utility. Additionally, while our multiomics approach provides comprehensive molecular characterization, functional validation through experimental models would strengthen mechanistic understanding of identified genes′ roles in tumor biology.

Future research directions include experimental validation of LRCD‐related genes′ functional contributions to tumor progression, investigation of their potential as therapeutic targets, and prospective clinical trials evaluating the prognostic model′s utility for treatment decision‐making. The integration of additional molecular features, such as epigenetic modifications and protein‐level data, could further refine prognostic accuracy.

## 5. Conclusion

In conclusion, this comprehensive multiomics investigation successfully established and validated a prognostic framework based on LRCD signatures in lung adenocarcinoma. Through integration of differential expression analysis, single‐cell transcriptomics, and machine learning approaches, we identified 14 prognostic genes and constructed a prognostic model with moderate‐to‐good discriminatory performance and meaningful clinical utility. The cellular heterogeneity revealed by single‐cell transcriptomic analyses provides novel insights into tumor microenvironment organization and immune‐tumor interactions. These findings advance our understanding of lung adenocarcinoma biology and establish a foundation for personalized therapeutic strategies, potentially improving patient outcomes through refined risk stratification and treatment selection.

## Author Contributions

Runzhe Wang and Qingtao Zhao contributed equally as co‐first authors, responsible for study design, data analysis, methodology development, and manuscript writing. Shicheng Liu, Lingxin Kong, Dahu Ren, Jianghong Yan, and Shuo Yang contributed to data curation, validation, and manuscript revision. Guochen Duan supervised the study, acquired funding, and finalized the manuscript. Runzhe Wang and Qingtao Zhao contributed equally to this work.

## Funding

This study was supported by the Key Research and Development Program of Hebei Province, No.22377790D.

## Disclosure

All authors have read and agreed to the published version of the manuscript and consent to its publication in this journal. All authors approved the final version of this manuscript.

## Ethics Statement

This study utilized publicly available, deidentified gene expression datasets (TCGA‐LUAD, GSE131907, and GSE72094), which are exempt from institutional review board (IRB) approval requirements under applicable regulations for secondary use of anonymized public data.

## Conflicts of Interest

The authors declare no conflicts of interest.

## Supporting Information

Additional supporting information can be found online in the Supporting Information section.

## Supporting information


**Supporting Information 1** Table S1: The 187‐gene lysosome‐related cell death (LRCD) candidate panel. This table lists the 187 candidate LRCD genes curated for the analysis. Category indicates the functional category. Note provides gene synonyms or annotation remarks. “In Figure 2C univariate set (30)” indicates whether the gene was included in the univariate Cox analysis shown in Figure 2C (*n* = 30). “HR direction (Figure 2C)” indicates the direction of the HR in that analysis. “In figure‐supported model set (21)” indicates whether the gene was included in the figure‐supported model gene set (*n* = 21); this set is not equivalent to the final model gene count reported in the main text. LRCD, lysosome‐related cell death; HR, hazard ratio.

**Supporting Information 2** Table S2: Candidate LRCD‐related genes assessed in the prognostic analysis. This table lists the 21 candidate lysosome‐related cell death (LRCD) genes evaluated in the prognostic analysis. Direction indicates the direction reported in the original candidate gene table. Figure references follow the original S2 numbering and should be checked against the final figure numbering. LASSO coefficients are not listed in this candidate‐gene table.  ^∗^For HMOX1, Table S1 lists the gene as not included in Figure 2C univariate set and reports its HR direction as “–”; its univariate Cox status therefore requires verification.

**Supporting Information 3** Table S3: Machine‐learning workflow implemented in this study. This table summarizes the procedures actually implemented in the provided code: TCGA‐LUAD data splitting, LASSO‐Cox feature selection, Ridge regression, ElasticNet, stepwise Cox, C‐index evaluation, risk stratification with Kaplan–Meier analysis, and external validation in GSE72094. Only implemented algorithms are listed. Nested cross‐validation, RSF, GBM, SVM, time‐dependent ROC, calibration curves, and DCA were not implemented and are therefore not included. GSE68465 was not included in this supporting table. LASSO, least absolute shrinkage and selection operator; CV, cross‐validation; C‐index, concordance index.

## Data Availability

The data that support the findings of this study are available in The Cancer Genome Atlas. (TCGA) at https://portal.gdc.cancer.gov/, reference number TCGA‐LUAD. These data were derived from the following resources available in the public domain: GSE131907 (Gene Expression Omnibus, GEO), https://www.ncbi.nlm.nih.gov/geo/query/acc.cgi?acc=GSE131907.
